# Severe phenotype in an apparent homozygosity caused by a large deletion in the *CFTR* gene: a case report

**DOI:** 10.1186/1756-0500-7-583

**Published:** 2014-08-30

**Authors:** Raisa da Silva Martins, Ana Carolina Proença Fonseca, Franklyn Enrique Samudio Acosta, Tania Wrobel Folescu, Laurinda Yoko Shinzato Higa, Izabela Rocha Sad, Célia Regina Moutinho de Miranda Chaves, Pedro Hernan Cabello, Giselda Maria Kalil Cabello

**Affiliations:** Laboratório de Genética Humana, Instituto Oswaldo Cruz, Fundação Oswaldo Cruz, Pavilhão Leônidas Deane sala 611, CEP: 21040-360 Avenida Brasil 4365, Rio de Janeiro, Brazil; Laboratório Interdisciplinar de Pesquisas Médicas, Instituto Oswaldo Cruz, Fundação Oswaldo Cruz, Rio de Janeiro, Brazil; Laboratório de Parasitologia, Instituto Conmemorativo Gorgas de Estudios de la Salud, Panama City, Panama; Departamento de Pneumologia, Instituto Nacional de Saúde da Mulher, da Criança e do Adolescente Fernandes Figueira, Fundação Oswaldo Cruz, Rio de Janeiro, Brazil; Departamento de Nutrição, Instituto Nacional de Saúde da Mulher, da Criança e do Adolescente Fernandes Figueira, Fundação Oswaldo Cruz, Rio de Janeiro, Brazil; Laboratório de Genética Humana, Universidade do Grande Rio, Unigranrio, Rio de Janeiro, Brazil

**Keywords:** Cystic fibrosis, *CFTR*, Apparent homozygosis, Large deletion, Severe phenotype, Brazilian patient

## Abstract

**Background:**

Over 1900 mutations have been identified in the cystic fibrosis conductance transmembrane regulator gene, including single nucleotide substitutions, insertions, and deletions. Unidentified mutations may still lie in introns or in regulatory regions, which are not routinely investigated, or in large genomic deletions, which are not revealed by conventional molecular analysis. The apparent homozygosity for a rare, cystic fibrosis conductance transmembrane regulator mutation screened by standard molecular analysis should be further investigated to confirm if the mutation is in fact homozygous. We describe a patient presenting with an apparent homozygous *S4X* mutation.

**Case presentation:**

A 13-year-old female patient of African descent with clinical symptoms of classic cystic fibrosis and a positive sweat test (97 mEq/L, diagnosed at age 3 years) presented with pancreatic insufficiency and severe pulmonary symptoms (initial lung colonization with *Pseudomonas aeruginosa* at age 4 years; forced vital capacity: 69%; forced expiratory volume: 51%; 2011). Furthermore, she developed severe acute lung disease and recurrent episodes of dehydration requiring hospitalization. The girl carried the *CFTR* mutation *S4X* in apparent homozygosity. However, further analysis revealed a large deletion in the second allele that included the region of the mutation. The deletion that we describe includes nucleotides 120–142, which correspond to a loss of 23 nucleotides that abolishes the normal translation initiation codon.

**Conclusion:**

This study reiterates the view that large, cystic fibrosis conductance transmembrane regulator deletions are an important cause of severe cystic fibrosis and emphasizes the importance of including large deletions/duplications in cystic fibrosis conductance transmembrane regulator diagnostic tests.

## Background

Cystic fibrosis (CF; Omin #219700), the most frequent, life-limiting autosomal recessive disorder among Caucasians, is caused by mutations in the cystic fibrosis conductance transmembrane regulator (*CFTR*) gene [1]. In Europe, the carrier frequency is 1:25, resulting in a disease incidence of 1 in 2500 live births [2]. However, this incidence is quite variable, with a range from 1/500 in Ohio Amish to 1/90000 in Hawaiian Orientals [3]. The *CFTR* gene is characterized by an extremely large number of mutations (more than 1900) [4], the most common being the *F508del*. Other disease-causing mutations are distributed throughout all regions of the world, often with very low frequencies, making the molecular diagnosis difficult, especially in countries outside the European axis where the *F508del* frequency is relatively low, as observed in countries where ethnic composition is not predominantly Caucasian. Brazil is a typical example where the molecular diagnosis of CF is difficult owing to its multi-ethnic characteristics with a significant African component.

The design of diagnostic tests available on the market is usually based on the mutation panel of European or North American populations, which is unsuitable for Brazil. Little is known about the incidence of CF in Brazil, but it does vary among different regions of the country [5–8]. Among the European countries that contributed to the current ethnic composition of our population, the *F508del* allele frequency is 71.8% in Germany, 51% in Italy, 53% in Spain, 57% in Poland, 58% in Slovenia, and 45% in Portugal; the latter is the main Brazilian Caucasian component [9, 10]. The migratory waves from different regions of Europe, the intense inter-ethnic gene flow and different proportions of mixing genes of Caucasian, African, and Amerindian origins may explain the variation in the prevalence of the *F508del* mutation among different Brazilian regions [11].

Over the past 10 years, the *F508del* mutation contributed 30% of CF alleles in the State of Rio de Janeiro and disease incidence was estimated at 1/6902 live births [12]. Given the current annual number of births of 220,603 (Records of the Ministry of Health of Brazil, 2011; http://www2.datasus.gov.br/DATASUS/index.php), approximately 32 patients are born with CF each year. Until now, mutation screening allowed us to identify 29 different mutant alleles in CF patients from Rio de Janeiro. However, the detection rate is only 60%, which means that 40% of alleles remain unknown.

Over 1900 mutations have been identified throughout the *CFTR* gene, including single nucleotide substitutions, insertions, and deletions. Unidentified mutations may still lie in introns or in regulatory regions, which are not routinely investigated, or in large genomic deletions, which are not revealed by conventional molecular analysis. The apparent homozygosity for a rare *CFTR* mutation screened by standard molecular analysis should be further investigated to confirm if the mutation is in fact homozygous. Apparent homozygosity can result from a mutation of one allele and the presence of a large deletion encompassing the location of the first mutation on the second allele [13].

We describe a patient carrying the *S4X* mutation (CM930095) in apparent homozygosity; further analysis revealed that it was heterozygous with a large deletion that includes nucleotides 120–142, which corresponds to a loss of 23 nucleotides coding the first exon of the *CFTR* gene. This deletion removes a region from position -12 to position 10, including codons 1, 2, 3, and 4 of exon 1.

## Case presentation

A 13-year-old female patient of African descent with clinical symptoms of classic CF, a positive sweat test (97 mEq/L; diagnosed at age 3 years), pancreatic insufficiency (PI), and severe pulmonary symptoms (initial lung colonization with *Pseudomonas aeruginosa* at the age of 4 years) had a forced vital capacity of 69% and a forced expiratory volume of 51%. Furthermore, the girl developed severe acute lung disease and recurrent episodes of dehydration requiring hospitalization.

## Methods

Informed consent in *CFTR* studies was obtained from the proband and her parents at the time of referral. Genomic DNA was extracted from peripheral blood by a Purelink Genomic DNA Mini Kit (Invitrogen, Carlsbad, CA, USA). The DNA was tested for a proper panel of *CFTR* mutations from Rio de Janeiro [6] and the *S4X* mutation in apparent homozygosity was identified by allele-specific polymerase chain reaction (PCR) amplification. The apparent homozygosity for a rare mutation and the absence of the *S4X* mutant allele in the maternal DNA raised the suspicion that it was a false homozygosity. To evaluate the presence of a large deletion encompassing the same sequence region, amplified products were cloned using the pGEM-T® Easy Vector System (Fitchburg WI, USA) following manufacturer recommendations and all plasmids were purified by QIAprep® Spin Miniprep Kit (Qiagen, GmbH, Hilden) . Purified plasmids were sequenced by Sanger protocol with the BigDye® Terminator v3.1 Cycle Sequencing Kit (Applied Biosystems, Foster City CA, USA) according to the manufacturer’s instructions. Single nucleotide polymorphism from intragenic markers (*TUB9*-*T854T*-*M470V*) were genotyped using TaqMan® allelic discrimination assays (Assay ID: C__59055676_10; C___3021356_10; C___3021372_10, respectively, Applied Biosystems). The TaqMan® genotyping reaction was amplified on an Applied Biosystems® 7500 Fast Real-Time PCR System.

## Results

**Figure 1:**
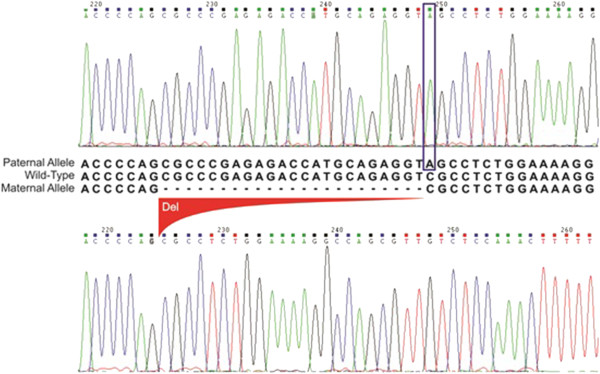
**Sequence comparison and alignment between the wt-CFTR sequence (middle capital letters) with the proband sequence eletropherograms.** Paternal chromosome (upper sequence) showing the change C to A corresponding to the *S4X* mutation; and maternal chromosome (lower sequence) with the 23 nucleotides deleted corresponding to the c.*120del23-CFTR* mutation.

The suspected presence of a large deletion in exon 1 was confirmed by cloning and sequencing analysis, performed on the DNA samples from both parents and the patient (Figure 1). This deletion removes 23 nucleotides from position 120–142, which affects the 5’ untranslated region upstream of exon 1, including the translation initiation codon (codons 133–135). This result confirms our initial suspicion that the patient was compound heterozygous for one rearrangement, instead of being homozygous for a rare allele. Analysis of intragenic markers showed that she was homozygous for the 2-1-2 (*TUB9*-*T854T*-*M470V*) haplotype.

## Discussion

When a rare mutation is detected in homozygosity in a molecular analysis the result should be confirmed. The distinction between real and apparent homozygosity has important implications for genetic counseling, prenatal diagnosis, and pre-implantation genetics. Whenever a molecular analysis identifies a rare mutation in the *CFTR* gene in apparent homozygosity, the patient should be tested for large deletions/duplications to avoid a false diagnosis. Until the true pathogenic nature of this *CFTR* mutation can be determined, the penetrance or segregation within the family and the effect on protein function cannot be confirmed with absolute confidence [13].

This case highlights the limitations of current molecular diagnosis tests based on PCR to detect large deletions in the heterozygous state. The patient experienced several episodes of lower respiratory tract infections with airway pathogen colonization (*P. aeruginosa, Staphylococcus aureus, Burkholderia cepacea* complex). She had insufficient pancreatic exocrine function with visible elimination of fat in feces and polyps in the gallbladder. In this case, the *S4X* in compound heterozygosity with *c.-12_10del23* results in a severe phenotype. Allelic frequencies of the *S4X* and *c.-12_10del23* for 618 CF chromosomes were 0.6% and 0.2%, respectively. The *120del23* mutation was first identified in two CF patients from the Azores [14]. Analysis of *CFTR* markers conforms with our previous findings about “risk” haplotypes associated with non-*F508del* mutations and by the intense gene flow among different ethnic groups that contributed to the Brazilian population composition [6, 15].

The *c.-12_10del23* mutation that we describe here was first detected in one Portuguese CF patient and submitted to the Cystic Fibrosis Mutation Database [4] by Ramalho and colleagues [12]. A PROVEAN score of −3.655 (cutoff = −2.5) for *c.1-10delATGCAGAGGT* confirms the deleterious characteristic for this deletion [16]. A possible effect is the activation of a potential downstream translation initiation site with a new reading frame, like that demonstrated by Ramalho and colleagues in subsequent studies to functionally characterize the effect of this mutation. They revealed that the *c.-12_10del23* mutation results in drastically reduced protein function and shows good correlation with the severe clinical CF phenotype [14]. Our observations are consistent with this finding, where the presence of two severe *CFTR* mutations leads to severe clinical features in the patient and suggests a reduced function of the protein. Other studies are necessary to better understand the association between these mutations and their pathogenesis.

## Conclusions

This study reiterates the view that large *CFTR* deletions are an important cause of severe CF and emphasizes the importance of including large deletions/duplications in the diagnosis of *CFTR* mutations when standard procedures reveal the presence of just one mutation, especially if that mutation is rare.

## Consent

Written informed consent was obtained from the patient’s legal guardian(s) for publication of this case report and any accompanying images. A copy of the written consent is available for review by the Editor-in-Chief of this journal.

### Ethics approval

The Ethics Committee of the Institute Oswaldo Cruz/Fiocruz approved this study.
